# Injuries in extreme sports

**DOI:** 10.1186/s13018-017-0560-9

**Published:** 2017-04-18

**Authors:** Lior Laver, Ioannis P. Pengas, Omer Mei-Dan

**Affiliations:** 1 Department of Trauma and Orthopaedics, University Hospital Coventry and Warwickshire, Coventry, UK; 2 Department of Trauma & Orthopaedics, Royal Cornwall Teaching Hospitals NHS Trust, Truro, UK; 3 CU Sports Medicine & Performance Center, Boulder, CO USA; 4 University of Colorado School of Medicine, Aurora, CO USA

## Abstract

*Extreme sports* (ES) are usually pursued in remote locations with little or no access to medical care with the athlete competing against oneself or the forces of nature. They involve high speed, height, real or perceived danger, a high level of physical exertion, spectacular stunts, and heightened risk element or death.

Popularity for such sports has increased exponentially over the past two decades with dedicated TV channels, Internet sites, high-rating competitions, and high-profile sponsors drawing more participants.

Recent data suggest that the risk and severity of injury in some ES is unexpectedly high. Medical personnel treating the ES athlete need to be aware there are numerous differences which must be appreciated between the common traditional sports and this newly developing area. These relate to the temperament of the athletes themselves, the particular epidemiology of injury, the initial management following injury, treatment decisions, and rehabilitation.

The management of the injured extreme sports athlete is a challenge to surgeons and sports physicians. Appropriate safety gear is essential for protection from severe or fatal injuries as the margins for error in these sports are small.

The purpose of this review is to provide an epidemiologic overview of common injuries affecting the extreme athletes through a focus on a few of the most popular and exciting extreme sports.

## Background

The definition of *extreme sports* (ES) inhabits any sports featuring high speed, height, real or perceived danger, a high level of physical exertion, and highly specialized gear or spectacular stunts and involves elements of increased risk. These ES activities tend to be individual and can be pursued both competitively and non-competitively [1]. They often take place in remote locations and in variable environmental conditions (weather, terrain) with little or no access to medical care [2], and even if medical care is available, it usually faces challenges related to longer response and transport times, access to few resources, limed provider experience due to low patient volume, and more extreme geographical and environmental challenges [3].

Examples of popular ES include BMX (Bicycle Motorcross) and mountaineering; hang-gliding and paragliding; free diving; surfing (including wave, wind, and kite surfing) and personal watercraft; whitewater canoeing, kayaking, and rafting; bungee jumping, BASE (*B*uilding, *A*ntenna, *S*pan and *E*arth) jumping, and skydiving; extreme hiking and skateboarding; mountain biking; in-line skating; ultra-endurance races; alpine skiing and snowboarding; and ATV (*A*ll-*T*errain *V*ehicle) and motocross sports [4].

In the last two decades, there has been a major increase in both the popularity and participation in ES, with dedicated TV channels, Internet sites, high-rating competitions, and high-profile sponsors drawing more participants [5–7]. The popularity of ES has been highlighted in recent years by the success of the X-games, an Olympic-like competition showcasing the talents in ES.

Participation in ES is associated with risk of injury or even death, and therefore, the extreme athlete—amateur or professional—and the medical personnel treating these athletes must consider the risk of injury and measures for injury prevention.

Recent data suggest that the risk and severity of injury in some ES is unexpectedly high [8].

Medical personnel treating the ES athlete need to be aware that there are numerous differences which must be appreciated between the common traditional sports and this newly developing area. These relate to the temperament of the athletes themselves, the particular epidemiology of injury, the initial management following injury, treatment decisions, and rehabilitation.

The purpose of this chapter is to provide an epidemiologic overview of the available literature on common injuries affecting the extreme athletes, the risk of their occurrence, and available prevention measures in this athletic population.

## Epidemiology of extreme sports injuries

Despite great evolution in traditional sports epidemiology, injury mechanisms in ES are less understood. Higher injury rates are seen in two groups: new and inexperienced athletes who have just started engaging in extreme sports and experienced extremists [4]. In some of these ES, we do not have a clear picture of the injury pattern due to lack of formal recorded events. What we do observe is an injury increase during competitions rather than training—a trend well recognized in common team sports [9, 10] as athletes are trying to push their limits even further for prizes, audience, or fame.

## Specific extreme sports and their associated injuries

### Skydiving

Skydiving is a major air sports of parachuting from an aircraft, the International Parachuting Commission (IPC) reported in 2009 approximately 5.5 million jumps, made by almost one million jumpers in 40 countries [11], including tandem jumps. The reported number of jumpers self operating their equipment added up to some 220,000 skydivers performing some 4.7 million skydives [12], with the majority of jumps being performed by a small number of skydivers whereas a larger number of participants perform fewer jumps [13–17].

Since the late 1980s, a few epidemiological studies have been conducted in order to establish the injury and fatality rates associated with the sport. Fatalities are seen more frequently in those who are considered “expert” or “seasoned” jumpers 60 vs. 20% with 71% occurring where the skydiver had at least one good parachute on, with the majority of fatalities (79%) been caused by human error [12].

Barrows et al. documented jumping incidents during two consecutive world free fall skydiving conventions in Illinois in 2000–2001 [13]. They followed 8976 skydivers making 117,000 skydives, in 20 days, indicating a total injury rate of 170 per 100,000 jumps while only 30% of those required a visit to an emergency department and as few as 10% continued to hospital admission. Most, 66% of the injuries were considered minor with 32% of these were abrasions and contusions and 22% lacerations. Of the jumpers who visited the emergency department for follow-up treatment, half suffered from extremity trauma which was related to lower extremity in 80% of patients with a rate of 0.5 fractures per 100,000 jumps.

Westman evaluated the skydiving injury rate during five consecutive years and more than half a million jumps in Sweden [18]. The incidence of non-fatal events was found to be 48 per 100,000 jumps (or 2100 jumps per incident as total or 3200 jumps per licensed jumpers), and 88% of those occurred around the landing with 51% of injuries involved the lower extremities, 19% involved the upper extremities, 18% involved the back and spine, and 7% involved the head, with 41% of the injuries categorized as minor, 47% as moderate, and 12% as severe. Most serious injuries were experienced by licensed skydivers while students in training had a six times higher injury rate. Interestingly, women over presented with injuries in this study, and they also had a higher proportion of landing injuries than men.

Although many parameters and participants may have changed over the last 20 years, injury rates remain similar. Modern equipment has decreased overall morbidity and mortality, but it has also led to faster landings with increased limb injuries.

### BASE jumping

**Fig. 1:**
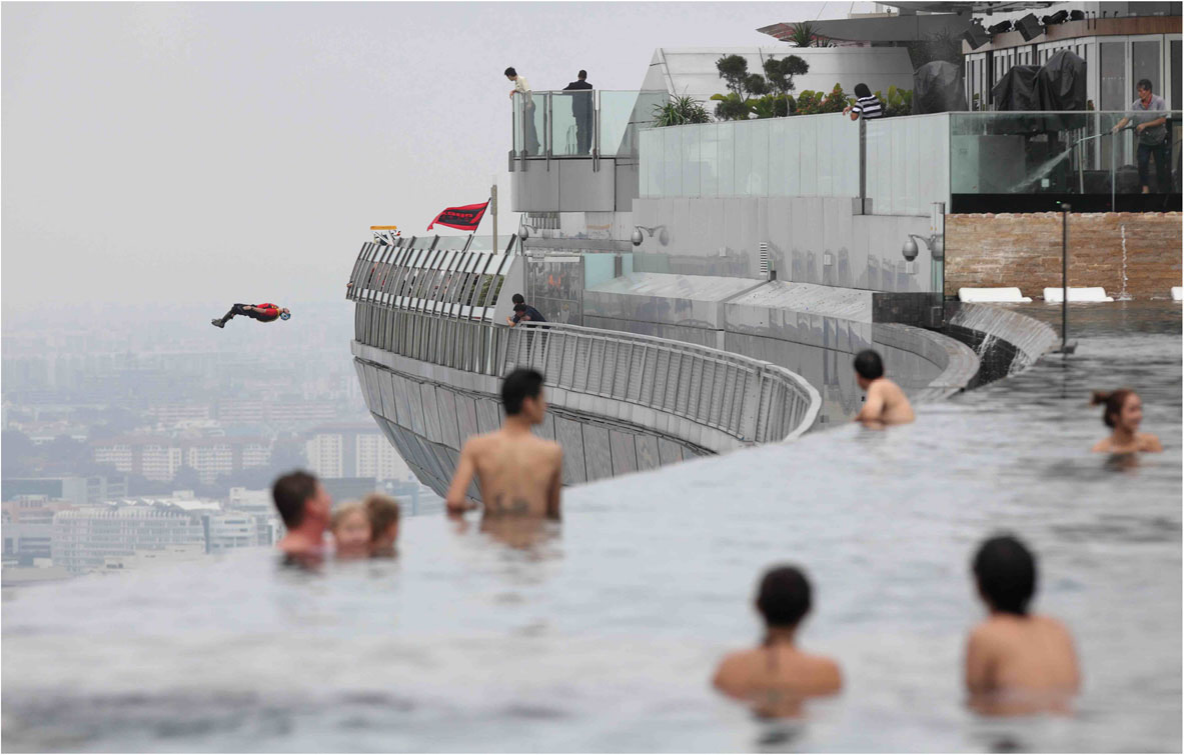
BASE jumping. With permission and courtesy of Omer Mei-Dan

BASE jumping (“BASE” stands for *B*uilding, *A*ntenna, *S*pan—a bridge, arch, or dome, and *E*arth—a cliff or other natural formation often less than 500 ft above ground level) has around 3000 active members; it is considered the most dangerous adventure sports in the world and a skydiving offshoot using specially adapted parachutes to jump from fixed objects (Fig. 1).

Very few studies have been conducted on this small unique population. Soreide et al. determined that BASE jumping is associated with a five- to eightfold risk for fatality or injury when compared to regular skydiving [19]. The fatality rate associated with BASE jumping was found to be 0.4 per 1000 jumps from a single site [19], although lacking information on demographic characteristics or jumpers’ experience level. In a study by Monasterio and Mei-Dan among 35 experienced BASE jumpers [20], an estimated injury rate of 0.4% was found in 9914 jumps, a finding similar to Soreide’s results [19]. Twenty-one (60%) jumpers in that study were involved in 39 accidents. The majority of accidents (28 accidents—72%) involved the lower limbs, 12 (31%) involved the back\spine, 7 (18%) the upper limb, and 1 (3%) was a head injury. It seems the sports attracts predominantly male participants. In Monasterio and Mei-Dan’s study, 75% of injuries were categorized as moderate or severe, as opposed Soreide’s series where most injuries were considered minor [20]. This could be explained by the fact that the single high site (1000 m) studied in Soreide’s series offered relatively safe jumping conditions allowing greater speed generation before parachute deployment and controlled landing. The rate of injuries requiring hospitalization in Monasterio and Mei-Dan’s study was 294 per 100,000 jumps and 16 times higher compared to the rate of such injuries in free fall skydiving found by Burrows et al. (18 per 100,000 jumps) [13]. A more recent study by Mei-Dan et al. analyzing fatality rates associated with wingsuit use in BASE jumpers showed a growing pattern of wingsuit-related fatalities, with 49% wingsuit-related fatalities between 2008 and 2011 and 90% in the first 8 months of 2013 compared to 16% between 2002 and 2007 [21]. Most fatalities occurred in the summer period in the northern hemisphere and were attributed to cliff or ground impact, being mostly the result of flying path miscalculations [21].

### Climbing

**Fig. 2:**
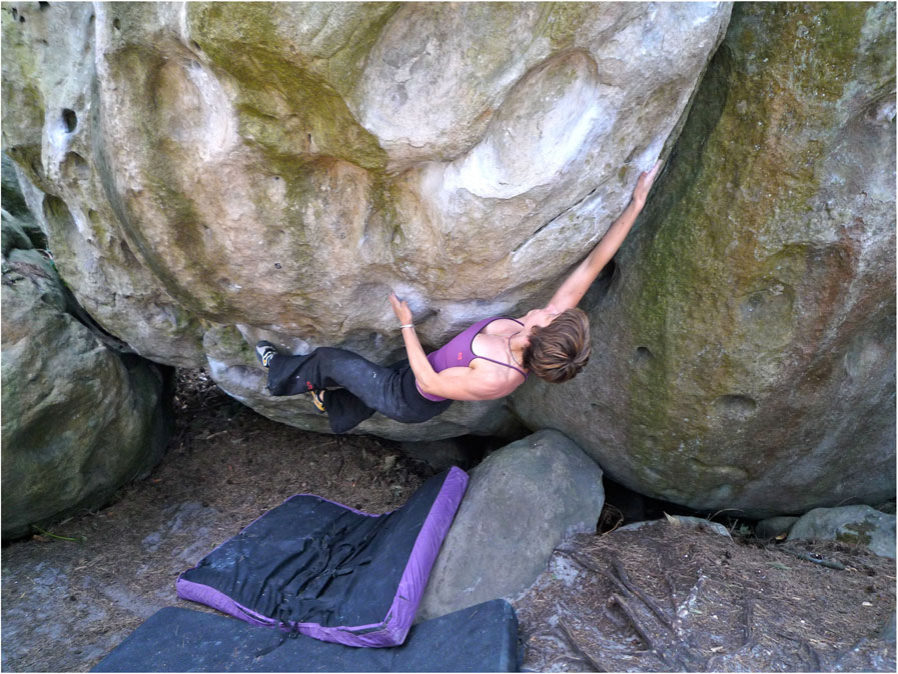
Bouldering. With permission and courtesy of Volker Schoffl

Climbing is an adventure sports which has developed from alpine mountaineering. Its popularity has vastly grown in the past three decades, with the introduction of indoor climbing gyms and climbing walls, becoming globally spread and evolved to new categories like ice climbing, bouldering, speed climbing, and aid climbing reaching an estimated two million participants in Europe and about nine million in the USA [22] (Fig. 2).

There are various disciplines encapsulated under the umbrella of climbing; some are less risky than others, with sports climbing or free climbing among the safest. A cross-sectional survey on rock climbing showed a lower frequency injury rate compared to football and horse riding [23], but with more catastrophic or fatal consequences.

Most studies show that the incidence of overuse injuries is associated with climbing frequency and difficulty [24, 25]. Most injuries are sustained by the lead climber, with falls being the most common mechanism of acute injuries [26]. Overall, most registered injuries in climbing studies are of minor severity. The fatality rate reported in climbing ranges from 0 to 28% climbers in various studies [27]. This wide range could be explained by varying methodology and data collection techniques in different series.

In indoor climbing, injury rates are much lower with 0.027–0.079 injuries/1000 h of participation and fatalities are very rare [28]. Overuse injuries are more common in this discipline, most commonly involving the upper extremities—mainly finger injuries. Although climbing relies on the synchronized and optimal function of the whole body, activity and performance are primarily limited by finger and forearm strength. Various gripping techniques lead to transmission of extremely high forces to the fingers, making overuse injuries of the fingers and hands the most common complaints in rock climbers [24, 25, 28–31]. Some injuries, such as flexor tendon pulley ruptures or the lumbrical shift syndrome, are very unique and specific for the sports and are rarely seen in other patient populations [24]. Very little data exists for ice climbing, and although severe injuries and fatalities occur, most recognized injuries are of minor severity and are comparable with other outdoor sports [27, 32].

Most studies on mountaineering report fatality/injury rates per 1000 climbers or 1000 summits, making it difficult to compare to the more common 1000 h of sports participation used in other disciplines. In mountaineering, additional environmental factors (avalanches, crevasses, altitude-induced illnesses with neurological dysfunction, etc.) can directly influence injuries and fatalities [33]. In high altitudes, it is important to also follow the prevalence of altitude illness, estimated between 28 and 34% above 4000 m [34, 35] and can be a major cause of injury, accident, or even death [36–38].

### Surfing

The sports of wave surfing is ever growing with a huge market involved, commercialization of surfing apparel and the surfing lifestyle, fashion trends, and media coverage. In 2009, it was estimated that there were more than 2.4 million surfers in the USA [39]. Despite being one of the most popular outdoor sports in the world, less than ten studies have been conducted on wave surfing.

Surfing is considered relatively safe compared to more traditional sports. A survey of self-reported injuries in Australia in 1983 found 3.5 “moderate to severe” injuries (resulting in lost days of surfing or requiring medical care) per 1000 surfing days [40]. The most common injuries requiring medical attention or resulting in inability to surf were lacerations (41%) and soft-tissue injuries (35%). A recent Australian survey found a rate of 2.2 significant injuries per 1000 surfing days [41] equating to 0.26 injuries/surfer/year, of those 45.2% were caused by collision with another surfer or surfboard. Distribution of lacerations, sprains, and contusions were similar to other reported rates, but they also reported 11% dislocation rate and 9% fractures.

Nathanson et al. evaluated acute competitive surfing injuries at 32 professional and amateur surfing contests worldwide between 1999 and 2005 [42]. The injury rate found was 5.7 per 1000 athlete exposures, or 13 per 1000 h of competitive surfing, with 6.6 significant injuries per 1000 h of competitive surfing. This injury rate compares favorably to those found in American collegiate football (33 per 1000 h), soccer (18 per 1000 h), and basketball (9 per 1000 h) where similar methods of data collection and injury definition were used [43]. The relative injury risk was calculated to be 2.4 times greater when surfing in waves overhead or bigger and 2.6 times greater when surfing over a rock or reef bottom.

In a Web site-based survey, 1348 individuals reported 1237 acute injuries and 477 chronic injuries [44]. Lacerations accounted for 42% of all acute injuries, contusions 13%, sprains/strains 12%, and fractures 8%. Thirty-seven percent of acute injuries were to the lower extremity, and 37% to the head and neck. Fifty-five percent of injuries resulted from contact with one’s own board, 12% from another surfer’s board, and 17% from the sea floor. This data correlates well with previous reports showing high incidence of lacerations caused by the sharp fin, the tail, or the nose of the surfboard. An interesting finding showed a considerable proportion of head injury, in contrast to the fact very few surfers use protective headgear [44, 45].

Fatality rates are unknown in surfing. Reports from Hawaii from 1993 to 1997 found that bodyboarders and surfers accounted for 17 of 238 ocean-related drownings [46]. This data includes fatal shark attacks. As 50% of a surfer’s time is spent paddling and 45% is spent remaining still, while only 3–5% is spent actually riding waves, most overuse injuries derive from paddling [47, 48]. Other data found overuse injuries to the shoulder (18%), back (16%), neck (9%), and knee (9%) [42].

Injury prevention in surfing is practiced by following basic safety recommendations such as maintaining adequate swimming skills (the ability to swim 1 km in less than 20 min and being comfortable swimming alone in the ocean) [49], familiarizing with the surfing environment and conditions (entry and points, currents, and underwater hazards), avoiding surfing to exhaustion, and safely practicing breath-holding training. Using adequate equipment is also essential such as temperature-appropriate wetsuits protecting against hypothermia, protected, rounded, and shock absorbing surfboard noses and fins trailing edges, and a board leash to keep the surfer’s board close at hand, and the board can be used as a flotation device should a surfer become exhausted or injured.

### Skiing and snowboarding

**Fig. 3:**
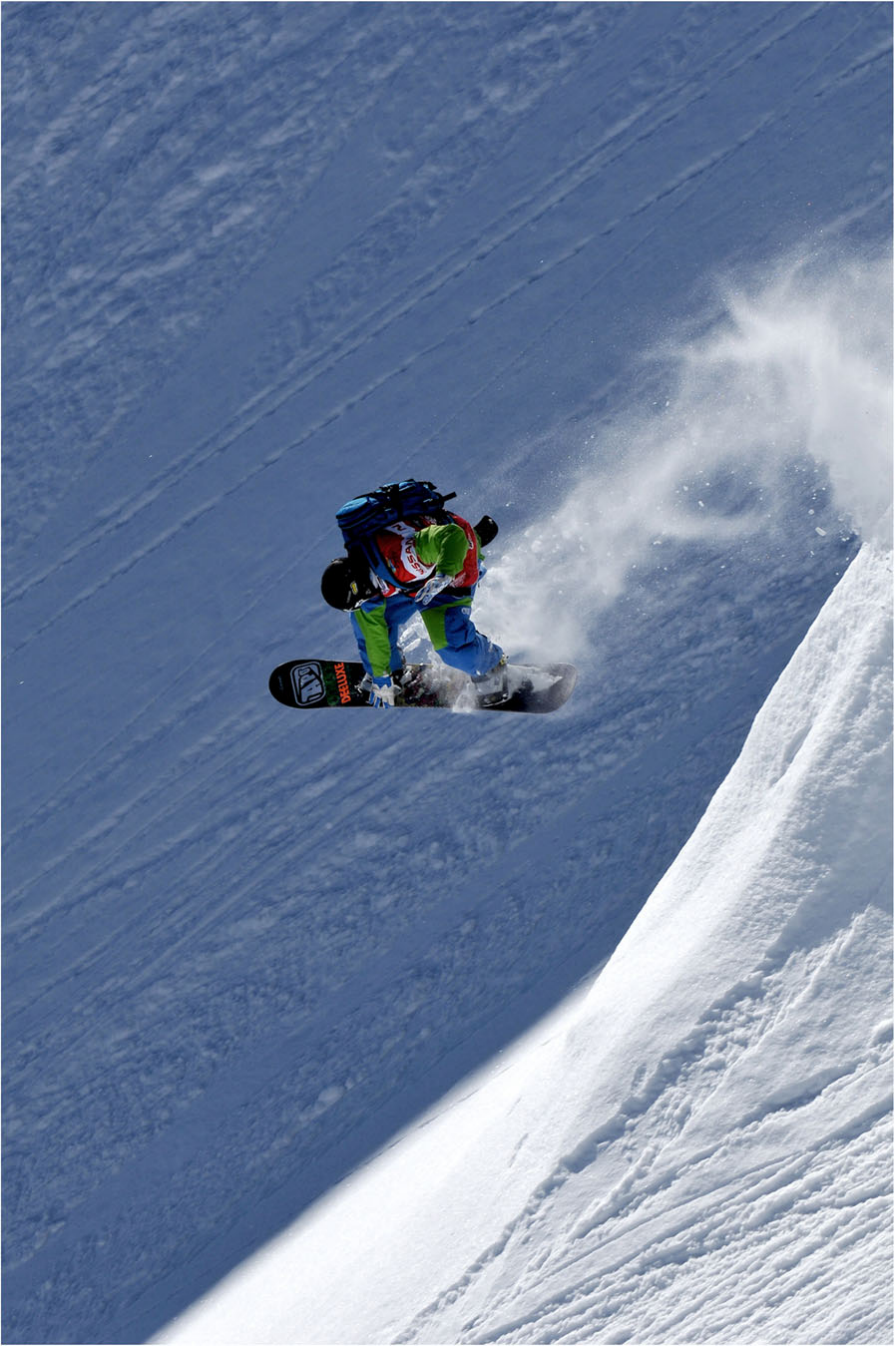
Snowboarding. With permission and courtesy of David Carlier

Skiing and snowboarding are the two main piste-based snow sports. With roots in Nordic (cross-country) skiing, Alpine skiing gradually evolved over time from method of transportation in Scandinavia thousands of years ago into the present recreational and competitive sport, becoming a winter Olympic sports in Garmisch in 1936, with snowboarding becoming an Olympic sports in 1998 (Fig. 3).

Although variable between resorts, currently approximately 60% of those on the slopes are Alpine skiers and 30–35% are snowboarders while the remainder perform ski boarding (snowblading) and Telemark skiing. Recent estimation report around 200 million skiers and 70 million snowboarders active in the world today.

The current risk of a recreational snow-sport-related injury is between 2 and 4 injuries per 1000 participant days [50], a risk much lower than in popular sports such as football and rugby, and has decreased steadily over recent years [51] thanks to improvements in equipment, ski area design and maintenance, and piste preparation [51]. The risk of injury from recreational Alpine skiing is generally accepted to be between 1 and 2 injuries per 1000 participant days [50, 52].

**Fig. 4:**
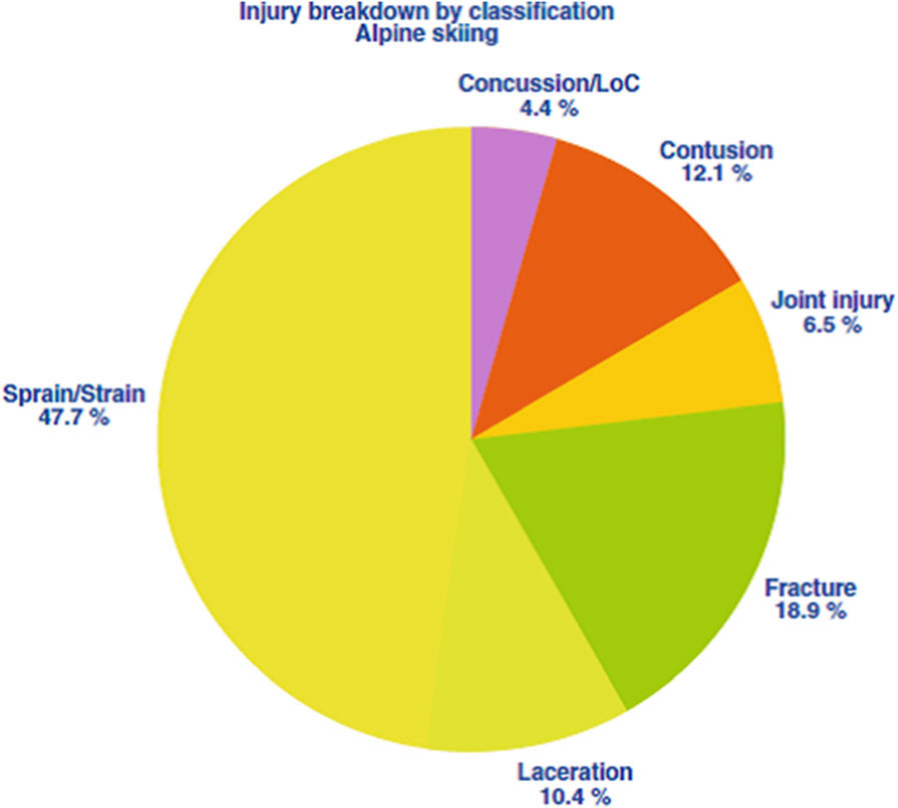
Injury types breakdown in Alpine skiing. From [69]. Used with publisher’s permission

**Fig. 5:**
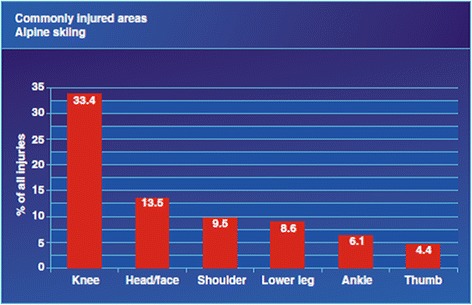
Commonly injured areas in Alpine skiing. From [69]. Used with publisher’s permission

The fracture rate from Alpine skiing is approximately 19% [53], and common sites include the clavicle, proximal humerus, and tibia. Prior to the introduction of release bindings, fractures of the lower leg were common from twisting forces transmitted unmitigated from the ski up to the lower leg. Even so, Alpine skiers are still more likely to injure their lower rather than their upper limb, with the knee joint being the single commonest site of injury among skiers, and most of these injuries are soft tissue/ligamentous in nature. (Figs. 4 and 5).

Upper limb injuries feature strongly with either the thumb or the shoulder being involved following a fall onto an outstretched hand. Thumb injuries almost exclusively affect Alpine skiers, so much so that the term “skier’s thumb” is used to describe the commonest injury—an acute radial stress to the metacarpophalangeal (MCP) joint of the thumb. The handle of the ski pole acts as a fulcrum across the MCP joint stressing the ulnar collateral ligament (UCL) [54]. If left untreated, this may lead to long-term functional disability.

The four commonest shoulder injuries to affect skiers and snowboarders are anterior dislocation of the glenohumeral joint, acromioclavicular (AC) joint disruption, clavicle fracture, and fracture of the proximal humerus. The incidence of shoulder injuries is higher in snowboarders [55].

The risk of injury from snowboarding is generally estimated at about twice that of Alpine skiing and currently stands at between 2 and 4 injuries per 1000 participant days [52]. Snowboarders are more likely to injure their upper limb than their lower limb [53]. Unlike skiers, when losing balance, snowboarders cannot step out a leg to regain balance. As a result, falls due to loss of balance are frequent, and not surprisingly, beginner snowboarders are at highest risk. This commonly results in falls on an outstretched hand and places the upper limb, and the wrist joint in particular, at high risk of injury [56]. The fracture rate among snowboarders is twice that of Alpine skiers [53], caused largely by the high rate of wrist fractures (up to 33% of all injuries [57].

**Fig. 6:**
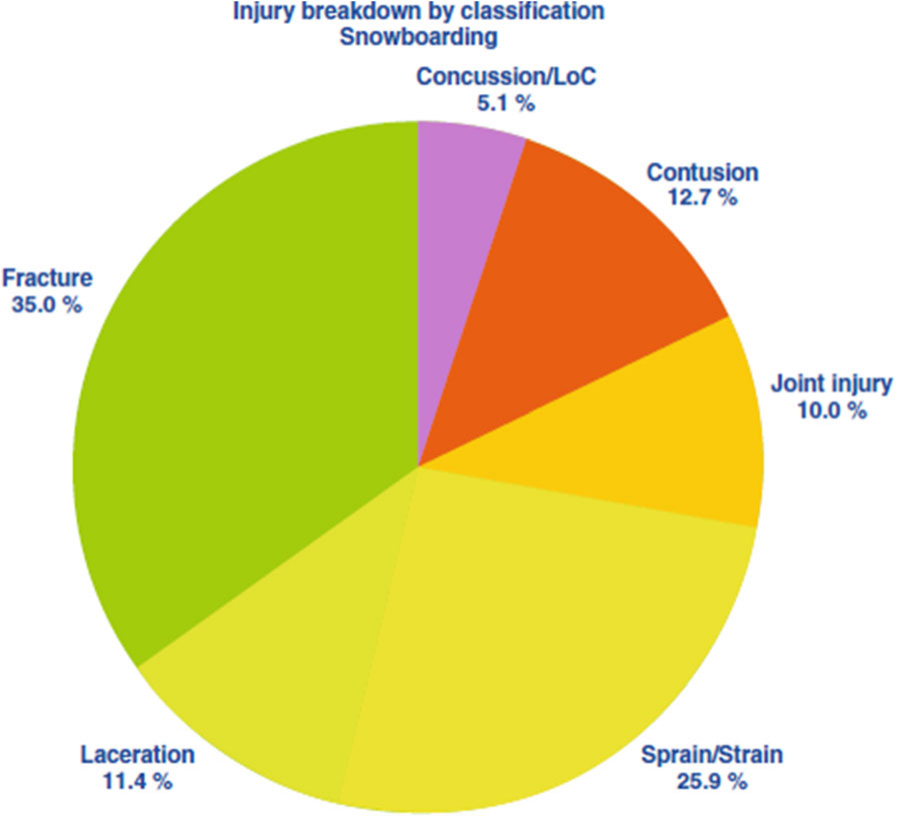
Injury types breakdown in snowboarding. From [69]. Used with publisher’s permission

Muscle and ligament strain/sprains are still common as are contusions from off-balance falls. Snowboarders suffer a higher rate of shoulder joint injuries due to an increased tendency to fall onto the upper limb [53]. Jumps and other aerial maneuvers, commonly performed in snowboarding, are associated with a relatively small but definite risk of injury to the spine [58–60]. Figure 6 illustrates injury types in snowboarding.

The injury risk among professional skiers and snowboarders is approximately three times that of recreational participants [61] and has been calculated to be 17 injuries per 1000 ski runs [62]. Almost one third of injuries among professional athletes were classified as severe, leading to an absence from participation of more than 28 days [63]. The knee is the commonest injury area among competitive skiers and snowboarders [61–63].

Knee injuries account for about one third of all skiing injuries. Most are minor soft tissue sprains. The medial collateral ligament (MCL) is commonly injured as a result of valgus force to the knee as the ski unintentionally splays the lower leg outward. Most grade 1 and 2 injuries will settle with conservative treatment. The most serious soft tissue knee injury involves the anterior cruciate ligament (ACL). This important ligament may be injured in isolation or in combination with other structures. While it is possible to ski without an ACL, this requires considerable effort and rehabilitation to maintain knee stability, muscle bulk, and proprioception. Most orthopedic surgeons recommend ACL reconstruction for those who wish to ski at or above an intermediate level. Knee injuries among snowboarders are much less common and usually result from direct trauma to the anterior aspect of the knee.

The fatality risk in snow sports is even lower at one death per 1.57 million participant days [64]. This equates to approximately 39 traumatic deaths per year in the USA out of a total of almost 60 million participant days (source: http://www.nsaa.org/). These fatality rates are much lower compared to other popular recreational activities such as swimming and cycling [64]. The commonest cause of a traumatic snow-sport-related death is a high-speed collision with a static object (tree, pylon, or another person) [65, 66]. Many of these deaths involve head injuries [66]. Non-traumatic causes of death on the slopes include ischemic heart disease, hypothermia, and medical events such as acute severe asthma attacks [65]. A less frequent but important mechanism of death is the so-called non-avalanche-related snow immersion death (NARSID), also known as a “tree well death” [66, 67], when skiers/snowboarders fall into a hidden pit underneath a tree. Unless the event is witnessed, self-extraction from the tree well is nearly impossible. The trapped individual tends to cause more snow to fall into the pit as they struggle to try to extract and death usually resulting from hypothermia or asphyxiation from snow falling in [68].

## Conclusions

Extreme sports are increasing in popularity, being fun to participate in and exciting to watch. The management of the injured extreme sports athlete is a challenge to surgeons and sports physicians. Appropriate safety gear is essential for protection from severe or fatal injuries as the margins for error in these sports are small. However, extreme sports athletes are more likely to return to their pre-injury levels of activity than the general population following treatment.
